# Chromosome-Level Genome Assembly and Comparative Transcriptome Analyses Identified Energy Conservation as a Key Strategy for Anadromous Adaptation of the Hilsa Shad, *Tenualosa ilisha* (Clupeiformes: Dorosomatidae)

**DOI:** 10.3390/biom15030321

**Published:** 2025-02-21

**Authors:** Kishor Kumar Sarker, Liang Lu, Roland Nathan Mandal, Md Rashedur Rahman, Anirban Sarker, Mohammad Abdul Baki, Chenhong Li

**Affiliations:** 1 Shanghai Universities Key Laboratory of Marine Animal Taxonomy and Evolution, Shanghai Ocean University, Shanghai 201306, China; kishorssf91@gmail.com (K.K.S.); llu@shou.edu.cn (L.L.); rashed739@gmail.com (M.R.R.); 2 Engineering Research Center of Environmental DNA and Ecological Water Health Assessment, Shanghai Ocean University, Shanghai 201306, China; 3 Key Laboratory of Freshwater Aquatic Genetic Resources, College of Fisheries and Life Science, Shanghai Ocean University, Shanghai 201306, China; roland.mandal322@gmail.com; 4 Department of Zoology, Jagannath University, 9-10 Chittaranjan Ave, Dhaka 1100, Bangladesh; anirban.sarker@zool.jnu.ac.bd (A.S.); mabaki@gmail.com (M.A.B.)

**Keywords:** hilsa shad, genome, migration, catabolism, LCPUFA, ELOVL2

## Abstract

Anadromous migration toward riverine tributaries is often challenged by altered environmental cues, food scarcity, and energy demands, sometimes at the expense of life itself. *Tenualosa ilisha* (Clupeiformes: Dorosomatidae), the national fish of Bangladesh, an anadromous shad, offers a model for understanding the molecular mechanisms of migration. To this end, we present a chromosome-level genome of *T. ilisha* and compare its transcriptomic imprints from muscle and liver across environments to trace the physiological shifts driving the migration. We observed rapid expansion of gene families to facilitate efficient signaling and osmotic balance, as well as a substantial selection pressure in metabolism regulatory genes, potentially relevant to a highly anadromous fish. We detected 1298 and 252 differentially expressed transcripts between sea and freshwater in the liver and muscle of *T. ilisha*, respectively, reflecting habitat and organ-specific adaptations. Co-expression analysis led us to hypothesize that the strength required for breeding migration toward upstream rivers is fueled by muscle protein catabolism forming ubiquitin-proteasomal complexes. In the liver, we observed a group of genes promoting fatty acid (FA) synthesis significantly in the riverine habitat. Regulation of FADS2 and ELOVL2 in the river reasoned the natural abundance of LC-PUFAs with better energy utilization in *T. ilisha*. Moreover, active gluconeogenesis and reduced insulin signaling in the liver are possibly linked to glucose homeostasis, potentially induced by prolonged starvation during migration. These genomic resources will accelerate the future evolutionary and functional genomics studies of *T. ilisha*.

## 1. Introduction

*Tenualosa ilisha* (Clupeiformes: Dorosomatidae), commonly known as hilsa shad, is the national fish of Bangladesh, valued for its significant ecological and economic importance. Due to its rich flavor and nutritional value, this fish is one of the most desired table fish and is treated as a symbol of cultural heritage. The geographic distribution of *T. ilisha* encompasses mainly the Bay of Bengal and the tributaries of the Ganges–Brahmaputra–Meghna river system [1,2,3]. Being an anadromous species (i.e., one that spends portions of its life cycle in freshwater for breeding and saltwater for feeding), covering distances up to 1200 km, it enters the freshwater ecosystem to secure successful breeding. However, the upstream breeding journey requires remarkable endurance and adaptability since the passage is challenged by varying osmotic pressure, geomagnetic alterations, hydrological transformation, and other niche constituents [4]. Anadromous species at their breeding grounds are often forced to compromise with food availability. For instance, Chinese tapertail anchovies undergo voluntary fasting and adopt an energy conservation mechanism during migration [5]. Similarly, Atlantic salmon adjust their metabolism rate to compensate for the increased energy requirements to accomplish the migration [6]. For Pacific salmon, a semelparous species (i.e., organisms that reproduce only once in their lifetime and then die), the cessation of feeding upon entering freshwater and the subsequent exhaustion of energy reserves lead to post-spawning mortality for both males and females [7]. Although the metabolic state or feeding behavior of *T. ilisha* during migration is not well documented yet, being a filter feeder, it likely encounters similar challenges due to inconsistent or desired prey unavailability in freshwater environments. This scenario suggests that *T. ilisha* must have evolved with a specialized adaptive system to survive in varying environments. Understanding the molecular mechanisms behind these adaptations is crucial for promoting the survival and reproductive success of *T. ilisha*.

Beyond its evolutionary significance and market value exceeding 4.0 billion USD, *T. ilisha* is notable for its unique biochemical profile, characterized by a high content of essential amino acids and unsaturated fatty acids (UFAs). These nutrients are vital for maintaining cell membrane fluidity and regulating energy during migration. From a human health perspective, the monounsaturated and polyunsaturated fatty acids (MUFAs and PUFAs) found in *T. ilisha* support brain development, reduce cholesterol levels, and contribute to cardiovascular health [8,9,10]. Transcriptomic analyses have further demonstrated a positive correlation between the upregulation of genes involved in long-chain PUFAs (LC-PUFAs) biosyntheses and the elevated production of these crucial fatty acids in riverine *T. ilisha* [11]. Despite their multifaceted significance, the genomic resources of *T. ilisha* are still limited and fragmented [12,13,14]. Until now, the best available genome was assembled within 2864 scaffolds by Mohindra et al. in 2019, and the absence of a chromosome-level genome assembly weakens a comprehensive understanding of the genomic features of *T. ilisha*. Moreover, existing studies have yet to provide any genome-wide comparison with sister taxa or detailed organ- and habitat-specific transcriptomic layouts to comprehend the key traits, including migration, energy budgeting, and lipid synthesis. It is worth noting that a recent population genetics study using exon capture has revealed that *T. ilisha* consists of a single panmictic population with low genetic diversity (F_ST_ = 0.001245 − 0.006612), posing a significant risk of being threatened [15]. Rapid changes in river morphology and the reliance on wild populations for nearly 600 K tons of annual capture further exacerbate this risk. From a conservation genetics perspective, *T. ilisha* is essential for preserving biodiversity and safeguarding the livelihoods of nearly three million people directly reliant on this species [16].

Hence, to determine the genetic loci associated with essential traits with higher precision, we outlined a project to produce the first chromosome-level genome assembly for *T. ilisha* by integrating Nanopore and Illumina sequencing with HiC reads. We aimed to identify the rapidly evolving genes and genes with selection pressure that facilitate survival in diverse environments by comparing the genome with its sister taxa. Furthermore, we compared the transcriptomic profiles of the liver and muscle tissues of *T. ilisha* collected from the river and sea to gain insight into migration biology, particularly the energy management strategies in varying environmental conditions. We hope this work will strengthen future genetic research, conservation efforts, and aquaculture initiatives for *T. ilisha*.

## 2. Materials and Methods

### 2.1. Sample Collection for Genome and Transcriptome

**Figure 1:**
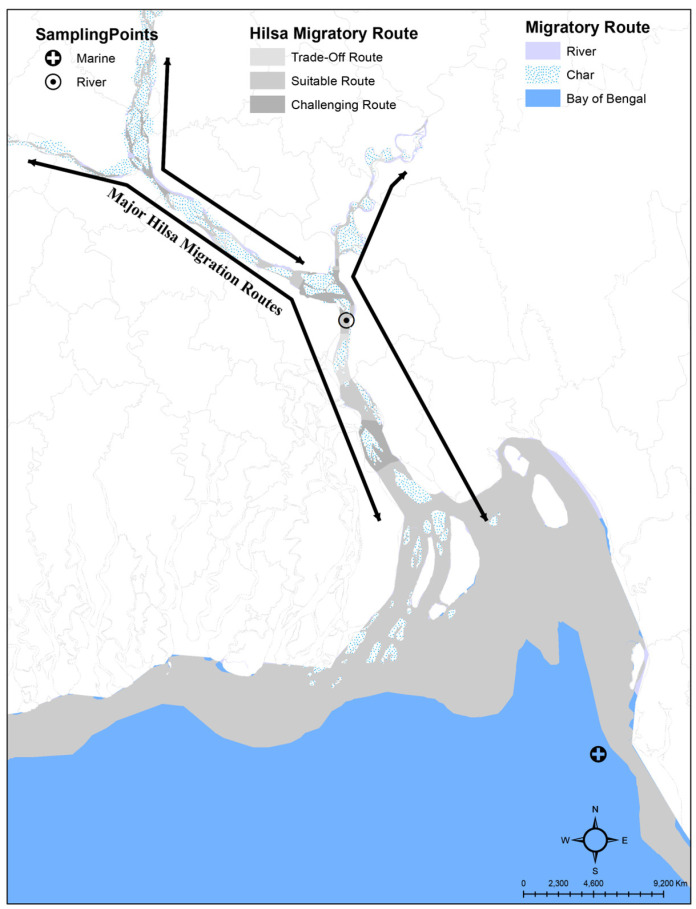
An Arc GIS map representing the migratory route of *Tenualosa ilisha* and the sampling collection points for the current study.

Live individuals of *T. ilisha* (Hamilton, 1822) were captured on board from both riverine (Meghna River: 23°02′15.5″ N 90°38′31.1″ E) and marine (Bay of Bengal: 21°27′14.5″ N 91°55′39.6″ E) habitats with the help of local fishermen (Figure 1). Due to the high oxygen demand and extreme stress sensitivity of *T. ilisha*, individuals died right after being captured. There were no further requisitions for euthanasia. Individuals captured more than six hours earlier were excluded from sampling due to concerns regarding RNA stability. Samples were obtained from nine different anatomical positions, encompassing the brain, blood, muscle, liver, kidney, heart, spleen, ovary, skin, spleen, and pyloric caeca using a single individual. Muscle tissues from a freshwater *T. ilisha* individual were selected for nanopore long-read sequencing. All samples were preserved in ALLProtect buffer (Beyotime, Haimen, Jiangsu, China, Cat No. R0121-100ml, Lot No. 042720200814). Blood samples were collected in EDTA anticoagulant tubes and stored at −80 °C [17]. Moreover, viewing the chromatin interactions, three live individuals were obtained in August 2023 from the Meghna River in the Chandpur district of Bangladesh (23°13′58.0″ N 90°38′20.8″ E). Liver and muscle tissues were promptly collected from the live fish, preserved in liquid nitrogen for two days, transported on dry ice, and stored at −80 °C for future analysis. Subsequently, the liver tissue of one specimen was sent on dry ice to Novogene, China, for Hi-C sequencing. Prior to sampling, individuals were subjected to morphometric and meristic counts to ensure correct taxonomic identification. The voucher specimen (FL_JNU_uncat. CH012312) was deposited in the Department of Zoology, Jagannath University, Dhaka, Bangladesh.

### 2.2. Sequencing of T. Ilisha Genome

The genome for *T. ilisha* was constructed using a third-generation sequencing platform (Oxford Nanopore Technologies, Oxford, UK) at the Genome Center of GrandOmics in Wuhan, China. High molecular weight (HMW) genomic DNA was extracted from muscle tissue using the CTAB method and purified with the QIAGEN^®^ Genomic kit (Cat#13343, QIAGEN, Hilden, Germany). DNA size selection (>10 kb) was conducted through the PippinHT system, and a 700 ng DNA library was constructed using the SQK-LSK109 ligation chemistry and sequenced on a Nanopore PromethION sequencer. Short paired-end reads (2 × 150 bp) with higher fidelity were generated for integration due to a considerable number of errors in long reads. Genomic DNA for the short reads was extracted using the Ezup Genomic DNA Purification Kit, and the library was prepared using 1 μg DNA and sequenced on an Illumina HiSeq10X platform [18,19]. For scaffolding the genome into chromosome level, Hi-C data were generated from the Illumina. A library (2 × 150 bp) was constructed from liver tissue preserved in all protect buffer according to a modified protocol by Belton et al. (2012) and sequenced on an Illumina Hiseq platform [20]. Quality control of raw reads was performed using HiC-Pro (v2.8.0). Low-quality reads (quality score < 20), adapter sequences, and reads shorter than 30 bp paired-end reads were trimmed off [21]. A total of 259,792,855 paired-end reads were kept for mapping against the assembled genome.

### 2.3. Chromosome Level Genome Assembly

The initial genome size of *T. ilisha* was estimated using Jellyfish with a k-mer size of 21 (-m 21), and subsequently, Genomescope was used to generate a histogram. The NECAT was utilized with parameters where the minimum read length was set to 3000 bp to assemble the Nanopore reads [22]. Since the assembly from long read is error-prone, the draft assembly was subjected to polishing. The NextPolish v1.4.1 program was used to polish the genome, mapping 131,504,324 Illumina reads with a maximum depth of 100 (-max_depth 100) [23]. We phased the polished assembly to create a haploid assembly and avoid regional duplication caused by heterozygosity within homologous scaffolds. To resolve this, the Purge Haplotigs pipeline was used to separate the genome into primary and pseudo haplotypes [24]. Minimap2 was included in the pipeline to align ONT reads to the assembled genome. Based on the coverage histogram, thresholds for low, mid, and high coverage were set to 11, 30, and 80, respectively (-l 11 -m 30 -h 80). The haplotype-resolved genome was then scaffolded to chromosome level using the 3d-DNA pipeline [25]. After indexing the genome and creating restriction enzyme cut site information (DpnII), the main program ‘run-asm-pipeline.sh’ was operated. The assembly was further rectified, primarily correcting misjoins using Juicebox. Finally, at each stage, we employed the Benchmarking Universal Single-Copy Orthologs (BUSCO) genes using the vertebrate database (-l vertebrata_odb10) to assess the assembly quality of the genome [26].

### 2.4. RNA Sequencing and Assembly

RNA extraction was performed using the TRIzol Reagent/RNeasy Mini Kit from Qiagen in Shenzhen, China. Following the extraction and quality control, double-stranded cDNA libraries were created from one microgram of RNA as per the manufacturer’s instructions. These libraries were purified, adapter-ligated, and subjected to PCR amplification using P5 and P7 primers. The PCR products were purified using beads, validated using Qsep100 from Bioptic (Taiwan, China), and quantified with a Qubit 3.0 Fluorometer from Invitrogen (Carlsbad, CA, USA). The prepared libraries were sequenced using Illumina Novaseq at GENEWIZ (Suzhou, Jiangsu, China), generating a total of 383,489,336 paired-end reads from nine tissue types of *T. ilisha* to annotate the genome, with over 90% Q30-passed reads. Subsequent processing involved fastp v0.12.4 to eliminate ambiguous reads and retain properly paired-end reads. Genome-guided transcriptome assembly of *T. ilisha* was performed using Trinity v2.15.1 [27]. Finally, all transcripts were clustered at 98% identity using CD-HIT-est v4.8.1.

### 2.5. Genome Annotation

RepeatModeler v1.0.4 was integrated with RECON, RepeatScout, and LtrHarvest/Ltr_retriever to identify transposable elements (TE) and their families in a non-model organism like *T. ilisha* [28]. The resulting de novo repeat library was then passed through the RepeatMasker v4.1.1 to quantify the repetitive elements (-e NCBI) [29]. For structural annotation, we employed the MAKER v3.01.03 pipeline utilizing two distinct approaches [30]. Firstly, homology-based prediction was conducted using assembled transcripts and the repeat library of *T. ilisha*, and annotated proteins of the Clupeiformes from NCBI. Secondly, taking the gene model from the initial round of annotation, ab initio prediction was performed using SNAP v2006-07-28 and Augustus v3.4.0. These results were then incorporated into the MAKER pipeline for a subsequent round of annotation. The resulting genes were aligned with public databases such as NCBI nr, KEGG, and GO. To identify protein motifs and domains, we employed InterProScan v5.51-85.0 by searching available repositories, including PANTHER, Pfam, PRINTS, and PROSITE [31]. Finally, genes were aligned against the genes of *Alosa alosa*, a species from the Alosidae family, which is the closest to the Dorosomatidae family, to identify homologous regions using MCScanX with default settings (--minspan = 10) (https://github.com/tanghaibao/jcvi/wiki/MCscan-(Python-version), accessed on 28 December 2024).

### 2.6. Gene Family Analysis

Gene family was inferred using proteins from the eleven species (*Alosa alosa*, *Alosa sapidissima*, *Chanos chanos*, *Clupea harengus*, *Coilia nasus*, *Danio rerio*, *Denticeps clupeoides*, *Engraulis encrasicolus*, *Sardina pilchardus*, *Tenualosa ilisha*, *Tenualosa thibaudeaui*). Only the longest isoforms from each species were taken to avoid redundancy. A comprehensive “all-by-all” blast was conducted to identify similarities among the proteins, and then the proteins were clustered into groups based on similarity index using the Markov clustering algorithm (MCL). From the final input, families with significant copy number variation were filtered out to reduce the noise. A maximum likelihood (ML) tree was constructed using 4252 single-copy orthologous genes employing Orthofinder v2.5.4 and IQtree v.2.3.6 software [32,33]. An ultrametric tree based on the ML tree was generated from r8s v1.81 software. The expansion and contraction of gene families were analyzed using CAFE, which leverages a stochastic birth and death process (λ) over the given ultrametric tree and number of gene families [34].

### 2.7. Positive Selection Analysis

A total of 4252 single-copy orthogroups from eleven species were carefully considered for the positive selection. The peptides from each orthogroup were aligned using MAFFT v7.475 with the ‘-linsi’ parameter [35]. The alignments were trimmed and backtranslated into nucleotides using trimAl v1.4.rev15 with the parameter ‘-automated1 -backtrans’. Gapped regions were then excluded using the ‘-nogaps’ parameter [36]. Sequences containing stop codons or those smaller than 60 codons were removed, resulting in 4216 sequences for subsequent analysis. The branch-site model (model = 2, Nssites = 2) with F3 × 4 nucleotide frequency was implemented using the codeml program from the PAML v4.9j suite [37].

### 2.8. Differential Expression Study

To investigate the expression difference reasoning migration, our study focused on muscle, liver, and blood, as muscle directly experiences locomotive stress, the liver acts as the metabolic center, and blood circulates metabolites throughout the body. The blood, liver, and muscle tissues from ten *T. ilisha* individuals, with five each from riverine and marine habitats, were sampled. After extracting RNA, blood samples did not meet the required replicate number (n = 5) to ensure robust statistical analysis. We excluded blood from further analysis, and muscle and liver tissues were sent for sequencing. The entire DE analysis was performed using scripts and programs from the Trinity-v2.15.1 package wrapped within a Docker environment. From the RNA-seq data, we performed combined assembly using the ‘Trinity’ program for two different tissue samples. The minimum transcript length was set to 200 bp (--min_contig_length 200), and the ‘--trimmomatic’ flag was kept open to remove Illumina adaptors. Following the assembly, transcript quality was evaluated, and abundance was quantified using the align_and_estimate_abundance.pl script (--est_method salmon). Expression estimation for each transcript in every sample was compiled into matrices, and cross-sample normalization was performed to generate transcripts per million (TPM) values for each of the transcripts using the abundance_estimates_to_matrix.pl script. Subsequently, edgeR was used to separate the differentially expressed transcripts. Finally, the transcripts were extracted, and a heatmap was generated to visualize differentially expressed transcripts [27,38]. Only transcripts exhibiting a 4-fold expression change at minimum FDR (*p* < 0.001) were retained for further analysis.

### 2.9. Co-Expression Analysis over DE Transcripts

A co-expression network was built for DE transcripts in the liver and muscle tissues of *T. ilisha* using the Weighted Correlation Network Analysis (WGCNA) package in R [39]. This involved computing a correlation matrix based on the TMM values of the transcripts, transforming it into an adjacency matrix, and identifying modules of highly correlated genes. A weighted signed network was computed with a soft power threshold of ‘7’ and ‘18’, respectively, for the liver and muscle. Hierarchical clustering was used to detect modules with gene clusters, which were visualized through topological overlap matrix (TOM) plots. The TOM plots and dendrogram helped to understand gene network interconnectedness and the clustering process. A topological overlap dendrogram defined the modules with a minimum gene size of 25 and 10 for liver and muscle, respectively. Furthermore, the correlation coefficient (KME) between module eigengenes (MEs) and traits was calculated to identify hub genes from the corresponding modules significantly associated with particular physiological traits or environmental conditions. Subsequently, Cytoscape v3.10 was utilized for further analysis and visualization of the network, while STRING was employed to explore protein–protein interactions within the significant modules [40].

### 2.10. Accession Numbers

All the processed raw sequencing data have been deposited in the NCBI repository (PRJNA1155700, PRJNA1147655). 

## 3. Results

### 3.1. Chromosome Level Genome Assembly and Annotation

**Figure 2:**
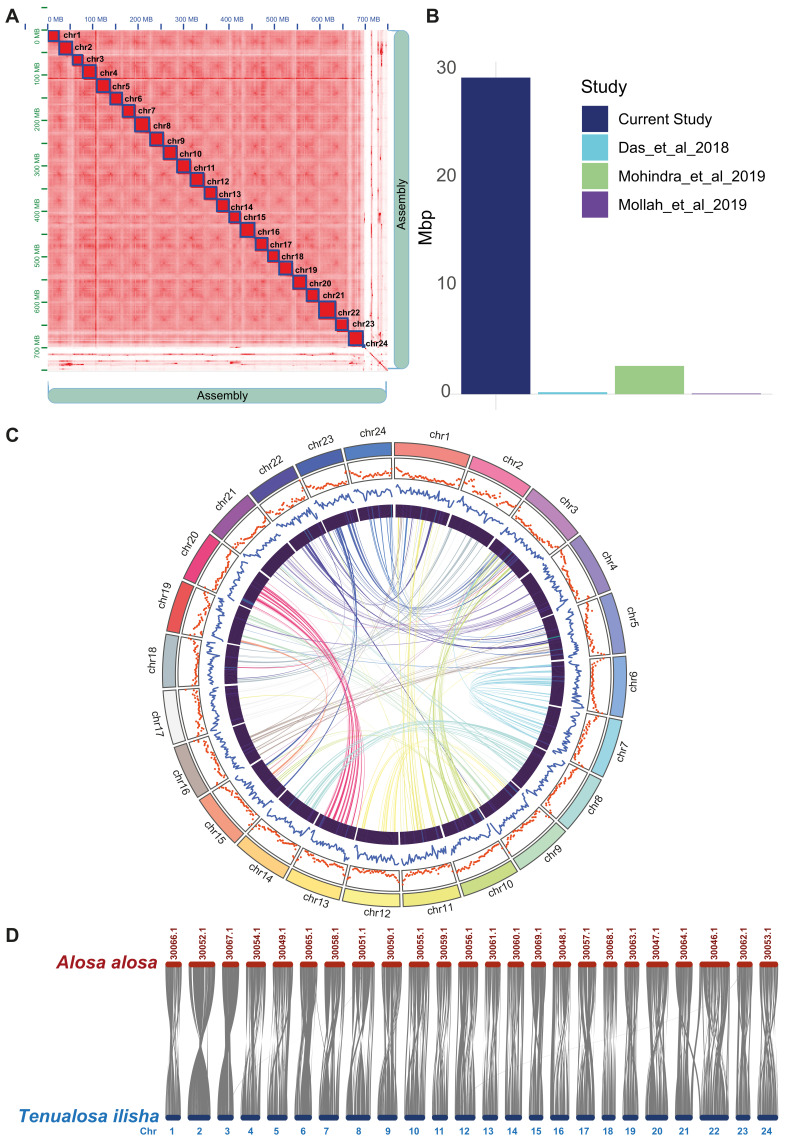
Genomic representation of *Tenualosa ilisha*. (**A**) The Hi-C heatmap shows the assembly of the *T. ilisha* genome. Each of the red square boxes represents each of the pseudochromosomes. (**B**) The bar plot compares the N50 between current and other studies [12,13,14]. (**C**) Circos plot presents the orientation and distribution of chromosomes, repeats, GC, genes, and the self-synteny from outside to inside direction. (**D**) Syntenic view of the assembled genome with a sister species, *Alosa alosa*.

By calculating the k-mer frequency distribution (k = 21), the genome size of *T. ilisha* was approximately estimated as 667 Mb. The analysis revealed a genome uniqueness of 83.9% and a heterozygosity rate of 0.641%, with a model fit reaching a maximum of 98.34% (Supplementary Figure S1). For a long contiguous genome, 47.39 Gb of Nanopore reads with 65× coverage were assembled, resulting in an initial assembly of 797 MB comprising 977 scaffolds with an N50 of 12 Mb. To correct potential sequencing errors introduced by the long reads into the draft assembly of *T. ilisha*, 40.19 Gb of short reads were aligned back to the genome. After polishing, the completeness of the genome was assessed using single-copy ortholog genes from the Vertebrata and Actinopterygii databases. The BUSCO score improved from 93.9% to 95.1% and 94.6% to 96.5% for the Actinopterygii and Vertebrata database, indicating a better assembly after polishing (Supplementary Figure S2). We removed alternate versions of the same genomic regions from the polished assembly to make the downstream analysis less complicated. Approximately 575 alternate haplotigs were discarded, accounting for 6.1% of the nucleotides. Finally, we used 104× coverage of Hi-C data to identify chromatin interactions and produce pseudochromosomes. Mapping the Hi-C reads against the genome resulted in a 748 Mb assembly, significantly increasing N50 to 29 Mb. Hi-C scaffolding anchored 93.03% of the linear assembly into 24 pseudochromosomes, with the largest pseudochromosome measuring 38.84 Mb (Figure 2A). This current assembly confirmed a significant improvement over the previous most contiguous *T. ilisha* genome with an N50 of 2.65 Mb (Figure 2B) [12]. The GC content of our assembled genome was 43.31%. The assembly statistics are presented in Supplementary Table S1.

In the *T. ilisha* genome, approximately 32.09% of the total genome size comprised repetitive elements. The major share of the interspersed repeats included 8.35% retroelements, 2.74% SINEs, 4.16% LINEs, and 4.82% DNA transposons. Additionally, simple repeats accounted for 6.29% of the genome, whereas 11.31% remained unclassified (Supplementary Table S2). From nine tissues, we assembled a total of 1,069,132 transcripts. These transcripts were clustered into 412,265 distinct transcripts for homology-based gene prediction. In addition to repetitive elements and transcripts, we compiled 531,831 protein sequences from 12 species, primarily from the Clupeiformes order. Structural annotation identified ~26,000 putative genes, with 91.5% having an annotation edit distance (AED) ≤ 0.5. Any gene below the AED threshold was discarded, and 24,258 genes were retained with high confidence. The distribution of protein-coding genes and repetitive elements throughout the pseudochromosomes is displayed in Figure 2C. Functional annotation was comprehensive, with 94.5% of genes annotated via InterProScan v5.51-85.0, 75.1% identified with Pfam domains, 61.9% assigned Gene Ontology (GO) terms, and 55% annotated through KEGG pathways. Finally, we examined the collinearity between the genomes of *T. ilisha* and its sister species, *Alosa alosa*, to assess the conservation of homologous genes. A total of 412 syntenic blocks were identified, with an average of 41 genes per block. The block with the highest gene count contained 418 genes, while the smallest block had 10 genes (Figure 2D). Notably, 82% of the genes of *T. ilisha* showed alignment with minimal chromosomal rearrangements across 24 chromosomes of *A. alosa* (Supplementary Figure S3). This high degree of synteny highlights the strong conservation of homologous genes within corresponding chromosomes and further supports the high quality of the current genome assembly (https://doi.org/10.6084/m9.figshare.26966197.v1, accessed on 2 February 2025).

### 3.2. Expansion in Gene Families

**Figure 3:**
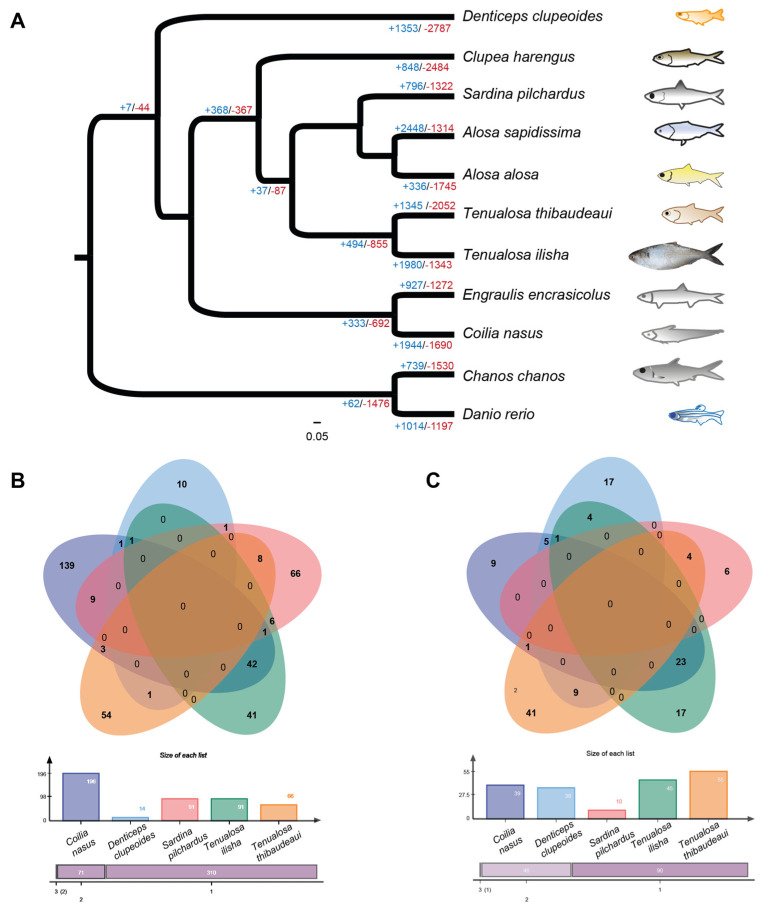
Representation of gene family expansion and contraction of *Tenualosa ilisha* and related species. (**A**) A phylogenetic tree based on 4252 one-to-one orthologous genes to infer the expansion and contraction of 11,849 gene families. The plus and minus signs refer to the expanded and contracted gene families. (**B**) A Venn diagram presents the rapidly expanded families, while (**C**) a Venn diagram presents the rapidly contracted families. For selecting species in the Venn diagram, we kept at least one migratory species, i.e., *Coilia nasus*, other than *T. ilisha*, one sole freshwater species, i.e., *Denticeps clupeoides,* and one sole marine water species, i.e., *Sardina pilchardus*.

From a pool of 287,064 proteins across 11 species, approximately 92% were clustered into 11,849 families with a significance level of *p* < 0.01. In *T. ilisha*, gene loss appeared to be more frequent than gene gain (+1345/−2052) (Figure 3A). Among 136 rapidly evolving gene families, 91 experienced expansions, while 45 underwent contraction. The SRCR domain-containing family (49) and the Fibronectin type III family (44) were seen to be expanded substantially. The rapid expansion of voltage-gated potassium channel genes (25) highlighted the potential significance of ion balance mechanisms required for hyper- and hypo-osmotic environments. The presence of genes in significant numbers from the Ankyrin repeat family (35) and EGF-like domain (24) underscored the importance of ion channels, integral membrane proteins, and related signaling mechanisms in long-distance anadromous journeys. Moreover, *T. ilisha* demonstrated a contrasting pattern of rapid expansion in at least 22 gene families compared to its freshwater sister clade, *T. thibaudeaui*, including families like ionotropic glutamate receptors (40*_t.ili_*/18*_t.thi_*), integrase catalytic domains (30*_t.ili_*/6*_t.thi_*), CCDC domains (28*_t.ili_*/3*_t.thi_*), SCAN domains containing transcription repressor or activator genes (10*_t.ili_*/0*_t.thi_*), and others, which are likely to contribute to its adaption on more diverse environment. Regarding rapidly evolving genes, even being phylogenetically distant, *T. ilisha* showed a similar pattern with migratory species like *Coilia nasus*, rather than more closely related species exclusively found in freshwater or marine environments (Figure 3B,C).

### 3.3. Evidence of Significant Positive Selection Across the T. ilisha Genome

We detected evidence of natural selection by estimating the ratio of non-synonymous (dN) to synonymous (dS) substitutions within protein-coding genes using codon models. An unrooted phylogenetic tree derived from 4252 orthogroups was constructed, with *T. ilisha* placed as the foreground species. Both the alternative (ω ≠ 1) and null (ω = 1) hypotheses were tested, and only those groups showing statistical significance (*p* < 0.05) in the likelihood ratio test (LRT) were retained as candidate genes. Our analysis identified 1353 genes under positive selection, providing strong evidence of significant positive selection across the *T. ilisha* genome. GO enrichment analysis revealed that the highest number of genes were linked to crucial functional categories; 312 genes were associated with metabolic processes (GO:0008152), 216 with catalytic activity (GO:0003824), and 448 with intracellular components (GO:0005622). Each category’s top 10 GO terms are presented in Supplementary Tables S3–S5.

### 3.4. Differential Expression of Transcripts in Liver and Muscle

In the current investigation, we conducted differential gene expression analyses in the liver and muscle tissues of *T. ilisha*. A total of 253,796 transcripts with an N50 of 1804 bp were identified in the liver, while the muscle tissue exhibited 272,368 transcripts with an N50 of 1827 bp. Considering the potential functional relevance, we retained low-expressed transcripts (TPM = 0) in our analysis. Our analysis revealed 53,174 and 55,261 transcripts having variations in expression from the liver and muscle tissues, respectively (Supplementary Figure S4A,B). Using a strict threshold of 4-fold change and a false discovery rate (FDR) of less than 0.001, we identified 1298 and 252 differentially expressed transcripts in the liver and muscle, respectively (Supplementary Figure S5A,B). We observed 259 upregulated transcripts in freshwater conditions and 1039 upregulated transcripts in marine conditions within the liver. Conversely, in the muscle tissue, 243 transcripts exhibited upregulation in freshwater, while only 9 displayed upregulation in marine conditions. This contrasting expression pattern in tissue types affirmed the tissue- and habitat-specific distinctive adaptive mechanisms of *T. ilisha*. Additionally, our Gene Ontology (GO) enrichment analysis over the DE transcripts unveiled significant enrichment of lipid transport (GO:0006869), lipid transporter activity (GO:0005319), lipid localization (GO:0010876), and acyltransferase activity (GO:0016747) in the liver. On the other hand, the catabolic process (GO:0009056) and intracellular protein-containing complex (GO:0140535) were notably enriched in muscle tissue, probably signifying the biological processes activated during the upstream journey.

### 3.5. Correlation of DE Transcripts

We conducted WGCNA to investigate the co-expression patterns of DE transcripts in the liver and muscle tissues of *T. ilisha*. Pearson correlation was applied to assess the strength of co-expression among genes and to evaluate the associations between modules or genes and phenotypes. A total of 1298 DE transcripts from the liver and 252 from muscle were considered for WCGNA.

**Figure 4:**
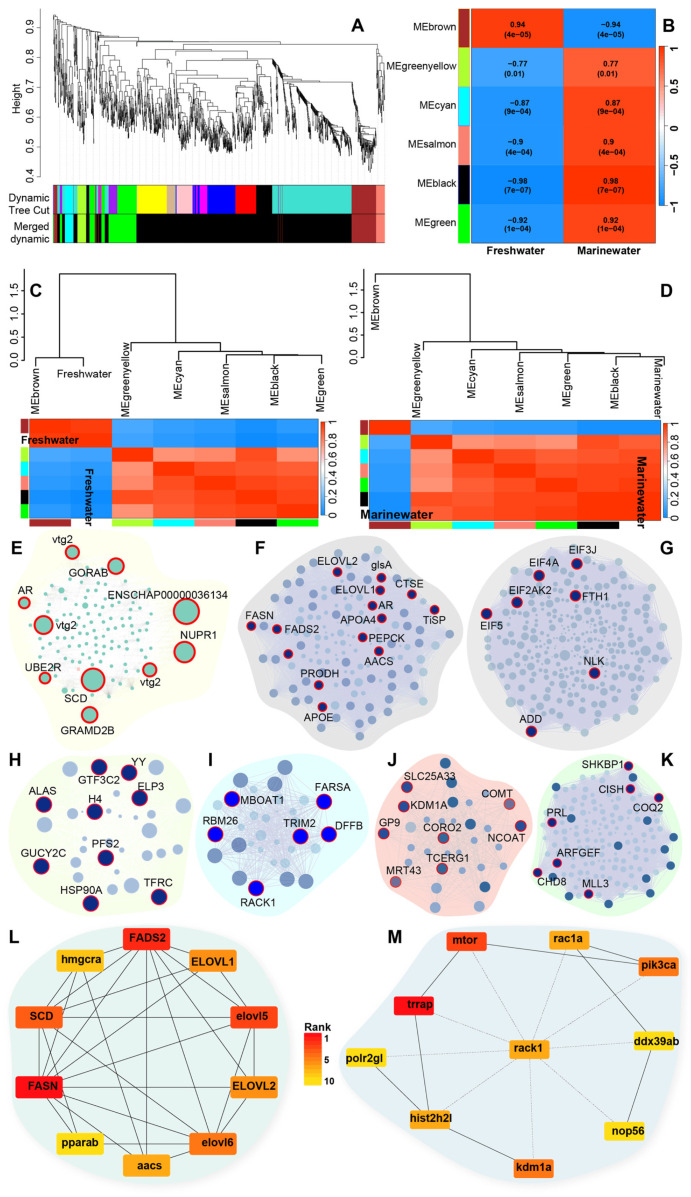
WGCNA Modules respecting liver transcriptome of *Tenualosa ilisha* for two distinct environments. (**A**) Cluster dendrogram showing module clustering results. The upper panel displays the hierarchical clustering dendrogram. In the lower panel, the colors show the module memberships determined by the methods on the left; (**B**) Module/trait relationships—Left panel of the Figure expresses correlation with the environmental phenotype by WGCNA analysis; right panel shows the gene significance to module membership in the six modules; (**C**) Clustering relationship of the freshwater environment with the significant color module based on module eigengene values; (**D**) Clustering relationship of marine water environment with the significant color module based on module eigengene values; (**E**) Co-expression network of freshwater upregulated genes under MEbrown module; (**F**) Co-expression network of freshwater upregulated genes under MEblack module; (**G**–**K**) Co-expression network of marine water upregulated genes under five significant modules; (**L**) STRING network of top 10 Hub genes with shortest biological paths for freshwater water environments; (**M**) STRING network of top 10 Hub genes with shortest biological paths for marine water environments.

In the liver, six highly significant modules were identified (Figure 4A–D and Figure S6): MEbrown (cor = 0.85, *p* = 2.3 × 10^−34^; transcripts no. = 119), MEblack (cor = 0.83, *p* = 1 × 10^−200^; transcripts no. = 925), MEcyan (cor = 0.54, *p* = 0.0012; transcripts no. = 33), MEsalmon (cor = 0.63, *p* = 8.5 × 10^−05^; transcripts no. = 33), MEgreen (cor = 0.68, *p* = 3.1 × 10^−22^; transcripts no. = 154), and MEgreenyellow (cor = 0.34, *p* = 0.049; transcripts no. = 34) (Figure S7A–F and Figure 4E–K). Among these, the MEBrown module was substantially correlated with the freshwater environment (Figure 4E). Within this module, key genes involved in fatty acid biosynthesis, such as SCD, FADS2, and ACSL, were highly connected. Additionally, vitellogenin genes (VTGs) were found to be upregulated and significantly connected within the module. Most of the genes from the MEBlack module were highly upregulated and correlated with the marine environment. A group of genes in the MEBlack module seemed to be influential for the marine environment when they were downregulated only (Figure 4F). These genes, such as very long chain fatty acid elongase (ELOVL1, ELOVL2), fatty acid synthase (FASN), and fatty acid desaturase 2 (FADS2 or ∆6), which are critical regulators of fatty acid biosynthesis and downregulated in marine environments, were found to be grouped into the MEBlack module. Furthermore, phosphoenolpyruvate carboxykinase (EC:4.1.1.32 or PEPCK), an enzyme involved in gluconeogenesis, was downregulated in marine water individuals and connected to genes controlling fatty acid synthesis.

**Figure 5:**
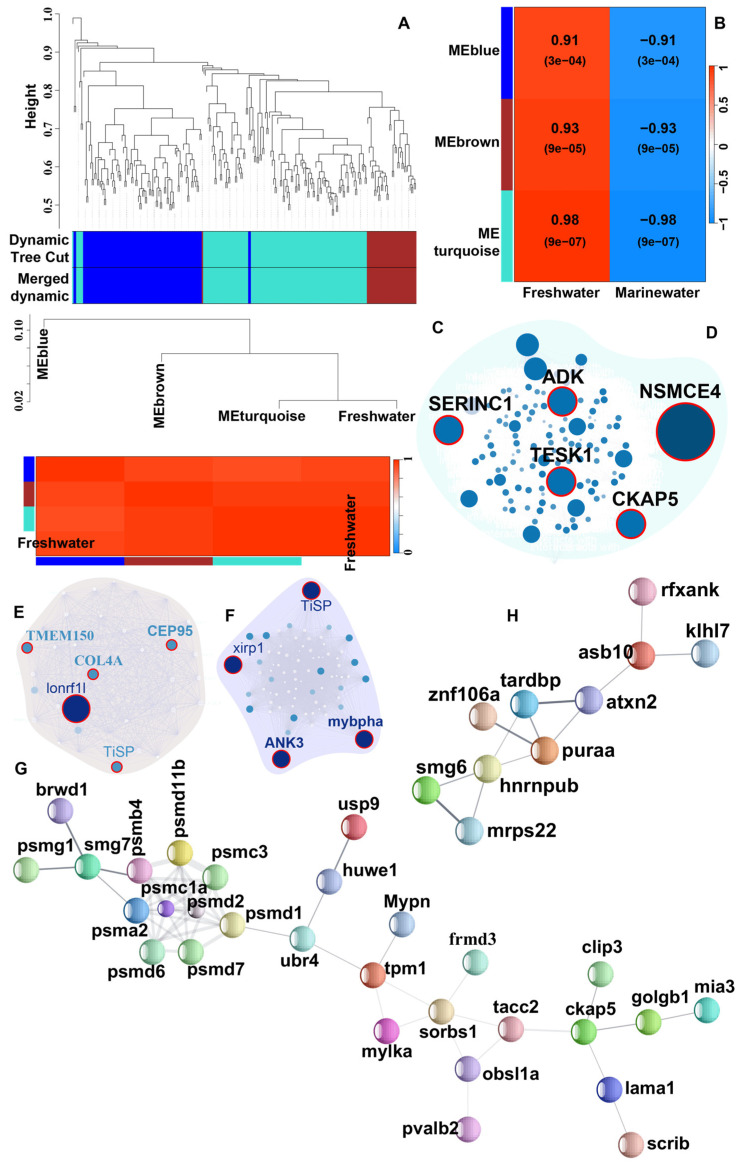
WGCNA Modules respecting muscle transcriptome of *Tenualosa ilisha* for freshwater environment. (**A**) Cluster dendrogram showing module clustering results. The upper panel displays the hierarchical clustering dendrogram. In the lower panel, the colors show the module memberships determined by the methods on the left; (**B**) Module/trait relationships—Left panel of the Figure expressed correlation with the environmental phenotype by WGCNA analysis; right panel shows the gene significance to module membership in the three modules; (**C**) Clustering relationship of the freshwater environment with the significant color module based on module eigengene values; (**D**–**F**) Co-expression network of genes under MEturquoise, MEbrown, and MEblue modules; (**G**) Cluster-1 of protein–protein interactions of STRING database for freshwater environments; (**H**) Protein–protein interactions of STRING database for freshwater environments.

For the muscle tissue, three significant modules were identified, all associated with the freshwater environment (Figure S10 and Figure 5A–C): MEblue (cor = 0.68, *p* = 1.2 × 10^−13^; transcripts no. = 88), MEbrown (cor = 0.7, *p* = 1.4 × 10^−06^; transcripts = 37), and MEturquoise (cor = 0.89, *p* = 2 × 10^−43^; transcripts = 124) (Figure S11A–C and Figure 5D–F). These modules were predominantly connected to the protein catabolic process. Several proteasomal proteins, including proteasome assembly chaperones (PSMG), proteasome 26S subunit ubiquitin receptor (PSMD), and proteasome 26S subunit (PSMC), were more active in freshwater conditions, indicating an accelerated protein degradation in muscle tissue of *T. ilisha*. Notably, several novel transcripts of *T. ilisha* were counted pivotal within these modules, probably pursuing the species–specific physiological necessity in a given environment. Moreover, we checked biological associations among the co-regulated transcripts from the liver and muscle tissues using the STRING database. We observed considerable associations among biologically linked transcripts in addition to the statistical connectivity (Figure S9A–E and Figure 5G,H). The top 10 interacting hub genes from each environment are presented in Figure 4L,M.

## 4. Discussion

Currently, there are three genome assemblies of *T. ilisha* with N50 values of 188 Kb, 64.16 Kb, and 2.65 Mbp, respectively [12,13,14]; all were outdistanced by the current assembly of this study featuring an N50 of 29 Mb. The improved assembly with rigorous annotations offered us the potential for comparative genome studies. Our comparative analysis of genomes revealed that *T. ilisha* has diverged from its sister taxa with adaptations supporting a migratory lifestyle characterized by an increase in genes related to signaling and osmoregulation. After scanning the orthologous genes, nearly one-third of them were identified under natural selection, primarily linked to metabolic processes. Moreover, gene expression analysis revealed tissue-specific functional roles in different environments. In muscle tissue, we noted a significant catabolic process during energy-demanding upstream migration. On the other hand, analyses of liver transcripts showed a decrease in protein synthesis rates in riverine habitats while lipogenesis intensified, particularly in the synthesis of long-chain polyunsaturated fatty acids (LCPUFAs) through the regulation of FADS2 and ELOVL2. This shift toward increased lipid synthesis appears to be an energy conservation strategy, optimizing reproductive efficiency amid the energy demands of migration and spawning.

Overall, this project not only delivers a comprehensive genomic resource but also lays a solid starting point for understanding the migratory biology of *T. ilisha*. Below, we will discuss the evolution of gene families and positively selected genes of *T. ilisha*, differential expression patterns in genes related to protein catabolism, biosynthesis of polyunsaturated fatty acids, and oocyte maturation, potentially crucial for anadromous adaptation.

### 4.1. Comparative Genomics and Adaptive Divergence of T. ilisha

Approximately 14 million years ago, *T. ilisha* diverged from its freshwater sister species, *T. thibaudeaui* [41]. Several gene families, including those with integrase zinc binding, CCDC, Laminin G, SCAN, IgV, and PNMA domains, have undergone rapid expansion in *T. ilisha* while contracting in *T. thibaudeaui*, indicating divergent evolutionary pressures shaped by differences in habitat and life cycle. Our analysis revealed a notable expansion of the ionotropic glutamate receptor (iGluR) family in *T. ilisha*, with 40 members, compared to 18 in *T. thibaudeaui* and 21 in the basal Clupeiform species *Denticeps clupeoides*. Even a solely marine species, *Sardina pilchardus*, has fewer iGluR members (22). The expansion of iGluRs, crucial for excitatory synaptic transmission, sensory processing, and cognitive functions such as learning and memory, seems to enhance the ability of *T. ilisha* to navigate between marine and freshwater environments and adapt to the physiological stressors of migration [42,43].

The epidermal growth factor (EGF) family is nearly doubled in size in *T. ilisha* compared to other freshwater or marine species studied. EGF signaling is vital for reproduction; in *Danio rerio*, loss of EGFR results in a complete arrest of follicle development [44,45]. The expansion of EGF family proteins in *T. ilisha* is expected to enhance ovarian development and function during spawning migration. Moreover, gene families associated with ion regulation, such as K channel proteins, expanded in *T. ilisha* but contracted in *T. thibaudeaui*, reflecting adaptations to the osmotic challenges during trans-environment mobility. Collectively, these findings highlight the role of gene family evolution in shaping the ecological and life-history adaptations of *T. ilisha*.

More than one-third of the orthologues genes (1353/4252) of *T. ilisha* showed evidence of positive selection. From enrichment analysis, we observed that genes under selection pressure predominantly performed catalytic activity, enzyme binding, and metabolism, likely to adapt to challenging migratory lifestyles where food availability varies considerably between riverine and marine environments. The selective pressures on metabolic pathways highlight the importance of finely tuned biochemical pathways that support efficient energy management, nutrient assimilation, and overall physiological resilience during these migrations.

### 4.2. Protein Catabolism in Muscle Backing the Long-Distance Journey

**Figure 6:**
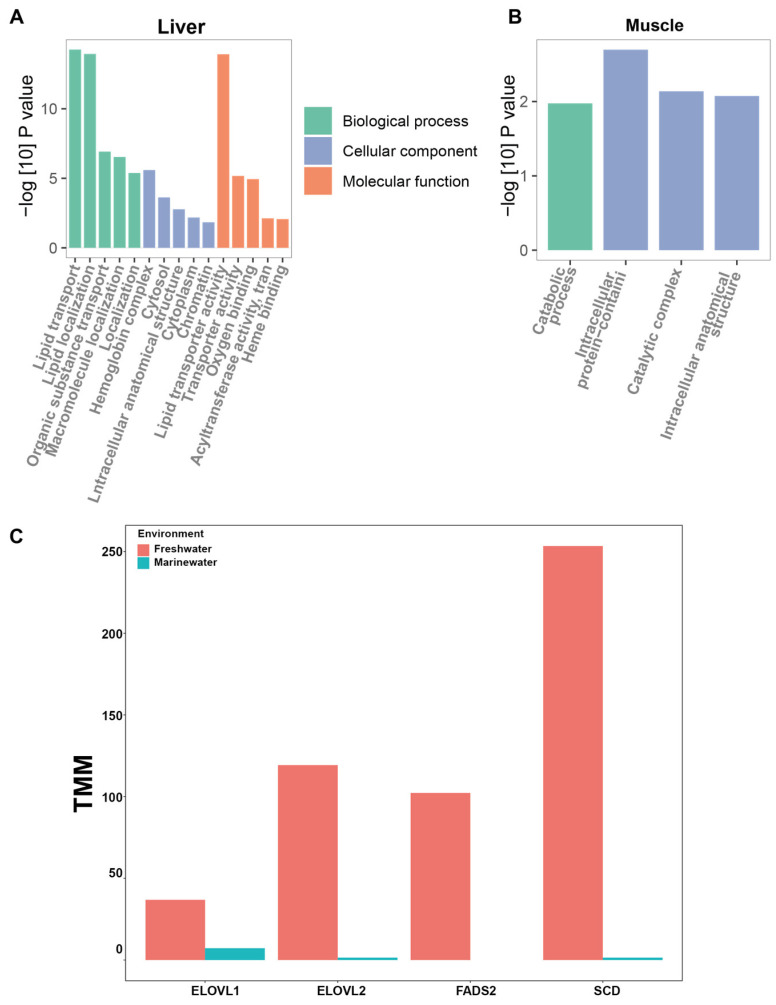
GO enrichment and selected gene expression in different tissues of *Tenualosa ilisha* in different environments. (**A**) Gene ontology term enrichment over DE transcripts from the liver; (**B**) Gene ontology term enrichment over DE transcripts from the muscle; (**C**) Expression differences of two FA elongases (ELOVL1 and ELOVL2) and two desaturases (SCD and FADS2) enzymes in marine and freshwater environments. Here, TMM refers to the trimmed mean of the M-values utilized for cross-sample normalization.

The WGCNA analysis over DE transcripts of the muscles revealed that nearly 95% of these transcripts were upregulated and correlated in individuals from riverine environments, and more importantly, they were biologically connected, also (Figure 5G,H). From GO enrichment analysis, we observed that a substantial proportion of these transcripts were associated with intracellular protein-containing complexes (GO:0140535) and catabolic processes (GO:0009056) (Figure 6B). Notably, the upregulated proteasomal proteins (PSMD1, PSMD2, PSMD6, PSMD7, PSMD11b, PSMA2, PSMB4, PSMC1a, PSMC3, PSMG1) in the muscle tissue of freshwater individuals appeared as biologically connected unanimously (Figure 5G). These proteins collectively form proteasomal complexes that degrade ubiquitin-tagged proteins, releasing amino acids that can be oxidized directly to generate ATP or converted into glucose through gluconeogenesis, eventually contributing to the energy supply chain [46,47]. In alignment with this assumption, we noticed the upregulation of Phosphatidylinositol-4,5-Bisphosphate 3-Kinase Catalytic Subunit Alpha (PIK3CA), an important element of PI3K pathway, which is expected to increase cellular glucose uptake in the freshwater individuals. We also observed significant co-regulation of biologically interconnected genes such as TRIM18, UBE2Q, HUWE1, HERC1, and CUL4 (Figure 5G). The activation of the entire proteasomal complexes suggested that muscle protein catabolism plays a crucial role in supplying the necessary energy needed for *T. ilisha* during the upstream migration. This observation connected the known patterns in fish, where catabolism is often intensified during migration, starvation, and gonad maturation, to support the associated energy-intensive activities [48,49]. In *Larimichthys crocea*, after two and half weeks of starvation, continuous protein degradation was observed once glycogen and fat reserves were depleted and energy demands were no longer met [50]. Similarly, an experiment in migratory *Oncorhynchus tshawytscha* with compromised feeding revealed amino acid catabolism and proteolysis in white muscle tissues [51].

Moreover, several essential genes from muscles were identified to provide further insights into the physiological adaptations of *T. ilisha* during migration. For instance, the upregulation of myosin-binding protein H (MYBPH) under riverine conditions regulates striated muscle contraction via interacting with actin filaments and myosin motors, suggesting enhanced muscular capacity and endurance to meet the increased physical demands of energy-intensive swimming [52,53] (Figure 5F). Cytoskeleton Associated Protein-5 (CKAP5) and Testis Associated Actin Remodelling Kinase-1 (TESK1) may further contribute to maintaining cellular structure and muscle resilience, while the upregulation of Collagen Type IV Alpha-3 Chain (COL4A) in riverine conditions points to active tissue repair and maintenance, essential for preserving muscle integrity under physical strain (Figure 5E).

### 4.3. Intensified Biosynthesis of Polyunsaturated Fatty Acids in River

In fish, lipid derivatives, like fatty acids, are essential for fish growth, reproduction, cold tolerance, and migration, providing metabolic energy alongside glucose and protein [54,55,56]. Fatty acids in fish can be synthesized de novo from non-lipid sources or obtained through dietary intake. Marine or cold-water fish, such as rainbow trouts, generally do not synthesize LCPUFAs independently due to their diet being naturally rich in these compounds. Conversely, freshwater or warm-water fish, like Murray cod, synthesize these internally due to lower dietary levels [57,58]. The synthesis rate of fatty acids inversely correlates with dietary lipid levels [59]. Anadromous species, like *T. ilisha*, exhibit an evolutionary advantage by inhabiting both marine and riverine environments. This adaptation of *T. ilisha* allows it to utilize diverse ecological niches and become a significant producer of unsaturated fats like docosahexaenoic acid (DHA) and eicosapentaenoic acid (EPA). It is widely accepted that riverine *T. ilisha* contains higher levels of essential fatty acids, contributing to their esteemed flavor and culinary value [60,61,62]. However, the genetic and metabolic pathways responsible for this have been largely unexplored.

Our WGCNA over DE transcripts identified key hub genes within the MEBrown and MEBlack modules (140 out of 925) that were consistently upregulated in freshwater environments. These correlated and biologically interconnected hub genes were linked to fatty acid elongation pathways (GO:0034626, GO:0034625, GO:0019367) (Figure 4L and Figure 6A). The key enzyme in fatty acid metabolism, FASN, was expressed at a 15 times higher level in the river. The key desaturase SCD (∆9) and FADS2 (∆6) were significantly upregulated within riverine individuals [63,64,65]. We identified three isoforms of FADS2 with over 100-fold increased expression at best compared to marine counterparts, proposing that desaturation predominantly occurs in freshwater environments, leading to a higher production of PUFA (Figure 6C). Several fatty acid chain elongation enzymes from the ELVOL family exhibited an increased level of transcription in riverine individuals. ELOVL2 exhibited over 80-fold higher expression in freshwater compared to marine environments and was sixteen times more active than ELOVL1 (Figure 6C). ELOVL1 elongates SFAs and MUFAs, while ELOVL2 is recognized for producing LCPUFAs, including omega-3 (EPA, DHA) and omega-6 (GLA, ETA) from PUFAs [66]. The pronounced activity of ELOVL2 in freshwater environments confirmed the reason for the rich LCPUFAs or omega-3 content in *T. ilisha*. The abundance of LCPUFAs can be attributed to their adaptation for a long migration with limited feeding opportunities. In general, fish prefer MUFAs over LCPUFAs as a substrate for β-oxidation [67,68]. LCPUFAs have a slower breakdown process than MUFAs and SFAs but yield more ATPs when metabolized [69]. This aligns with our finding that for energy during migration, *T. ilisha* may rely on lipid reservoirs, particularly LCPUFAs.

Additionally, we observed the upregulation of gluconeogenic genes, particularly PEPCK, in the MEBlack module, which is significantly correlated with fatty acid-related genes (Figure 4F). This upregulation indicated that glucose was synthesized from non-glucose substrates due to limited food availability in the river. Concurrently, key insulin signaling genes such as IRS1, mTOR, and PIK3CA were downregulated, indicating reduced glucose mobilization (Figure 4M). Interestingly, several eukaryotic translation initiation factors (EIFs) indicated a significant reduction in protein synthesis in the liver within the riverine environ-ment (Figure 4G and Figure S9). Generally, synthesizing proteins compared to lipids from glucose sources requires four times more ATP. In contrast, generating a single lipid molecule from acetate sources costs approximately eight times more ATP [70]. Therefore, producing lipids like FAs from glucose derivatives may be more sur-vival-friendly for *T. ilisha*. This metabolic adaptation supports the hypothesis that *T. ilisha* faces starvation in freshwater environments and relies primarily on lipid sources for energy.

### 4.4. Molecular Traces of Breeding Mechanism in River

Our study has provided insights into the co-regulation of genes associated with oocyte development and lipid metabolism in *T. ilisha* within the riverine environment. Notably, the MEBlack module from the liver revealed that zona pellucida-3 (ZP3) and VTGs genes, along with apolipoproteins (APOE, APOA), were upregulated and co-expressed (Figure S8 and Figure 4E,F). ZP3, crucial for oocyte and gamete development, exhibited a remarkable >4500-fold increase in expression compared to marine water, indicating its pivotal role in rivers during breeding. Similarly, VTG3, with a >2000-fold increase, reinforces the necessity of freshwater for oocyte maturation and fertilization in *T. ilisha*. Moreover, we observed upregulation of 3-hydroxy-3-methylglutaryl-coenzyme A reductase (HMGCRA) in the liver, a rate-limiting enzyme for cholesterol synthesis that is biologically connected directly with fatty acid synthesis genes and APOEs (Supplementary Figure S8). Apolipoproteins are high-density lipoproteins in vertebrates, playing a key role in regulating cholesterol levels of peripheral tissues via reverse cholesterol transport and contributing to gametogenesis and embryonic development [71]. From here on, the findings provide a molecular basis for the physiological adaptations required for successful reproduction in freshwater, highlighting the intricate regulatory mechanisms of lipid metabolism and oocyte maturation during the anadromous migration of *T. ilisha*.

## 5. Conclusions

Current chromosome-level genome assembly and detailed transcriptomic analysis from the muscle and liver tissues provide valuable insights into our understanding of the migration physiology of *T. ilisha* and offer a foundation for future research into the molecular basis of fish migration. It seems that in energy utilization, *T. ilisha* has evolved a distinctive strategy. Muscle protein degradation provides the necessary energy for swimming and migration. On the other hand, protein synthesis is turned down in the liver, likely to minimize the additional ATP expenditure. In fatty acid synthesis, it also prefers producing LCPUFAs, probably considering its long storage time and ability to generate more energy.

## Data Availability

The HiC scaffolded Genome was submitted to Figshare (https://doi.org/10.6084/m9.figshare.26966197.v1, accessed on 2 February 2025). Necessary supplementary information has also been deposited to Figshare (https://doi.org/10.6084/m9.figshare.26966137.v1, accessed on 2 February 2025).

## Supplementary Materials

The following supporting information can be downloaded at: https://www.mdpi.com/article/10.3390/biom15030321/s1, Figure S1: The GenomeScope plot shows the distribution of 21-mer depth for NGS reads of *Tenualosa ilisha*. The x-axis displays the coverage depth, while the y-axis indicates the count of k-mers observed at each depth; Figure S2: The bar plot represents the BUSCO percentage of *Tenualosa ilisha* against the vertebrate databases; Figure S3: The dot plot represents syntenic blocks shared between *Tenualosa ilisha* and *Alosa alosa*; Figure S4: Volcano plots representing transcripts with expression differences in the liver (A) and muscle (B) of *Tenualosa ilisha*. Red dots indicate transcripts with significant expression differences, while black dots represent non-significant transcripts; Figure S5: Heatmap showing differentially expressed transcripts in the liver (A) and muscle (B) of *Tenualosa ilisha* with a log 4-fold change and a false discovery rate (FDR) of less than 0.001; Figure S6: Network heatmap for all DE transcripts from liver of *Tenualosa ilisha* taken for WCGNA analysis; Figure S7: Module membership vs. gene significance for liver transcripts of *Tenualosa ilisha*; Figure S8: String network connecting FA synthesis pathway with developmental associate genes in liver of *Tenualosa ilisha*; Figure S9: (A–E) Protein-protein interactions of STRING database for marine water environments in liver of *Tenualosa ilisha*; Figure S10: Network heatmap for all DE transcripts from muscle of *Tenualosa ilisha* taken for WCGNA analysis; Figure S11: Module membership vs. gene significance for muscle transcripts of *Tenualosa ilisha*; Table S1: Assembly statistics of *Tenualosa ilisha* genome; Table S2: Repeat statistics of the genome of *Tenualos ilisha*; Table S3: Top 10 enriched GO terms over the positively selected genes (Biological process) in *Tenualosa Ilisha*; Table S4. Top 10 enriched GO terms over the positively selected genes (Cellular component) in *Tenualosa ilisha;* Table S5. Top 10 enriched GO terms over the positively selected genes (Molecular function) in *Tenualosa Ilisha.*

## Significance Statement

Beyond their delicacy and economic significance, the hilsa shad (*Tenualosa ilisha*) is an excellent model for studying fish migration. To understand the genetic basis of the anadromous lifestyle, we assembled a chromosome-level genome and analyzed tissue-specific transcriptomic differences across environments. Gene families like glutamate receptors and K^+^ channels rapidly evolved, likely in response to adopting an anadromous lifestyle. To support long-distance migration, riverine individuals prioritize LC-PUFA synthesis in the liver, involving FADS2 and ELOVL2, due to their slow oxidation rate and high energy yield. Starvation-induced energy deficits during breeding migration were tackled by simultaneous gluconeogenesis and reduced mobilization of insulin receptors. The energy conservation was supplemented by reduced protein synthesis. The ubiquitin-proteasomal catabolic process in muscle was pronounced to supply ATPs and subsequent muscular strength for migration. These energy management strategies highlight the remarkable adaptations that sustain the migratory lifestyle of *T. ilisha*.
