# Supplementary material for: Chromosome-Level Genome Assembly and Comparative Transcriptome Analyses Identified Energy Conservation as a Key Strategy for Anadromous Adaptation of the Hilsa Shad, Tenualosa ilisha (Clupeiformes: Dorosomatidae)
Source: Biomolecules. 2025 Feb 21;15(3):321. doi: 10.3390/biom15030321 (PMC11940632; doi:10.3390/biom15030321)
Supplement: Supplementary file 1 [file biomolecules-15-00321-s001.zip › biomolecules-3449630-supplementary.pdf]

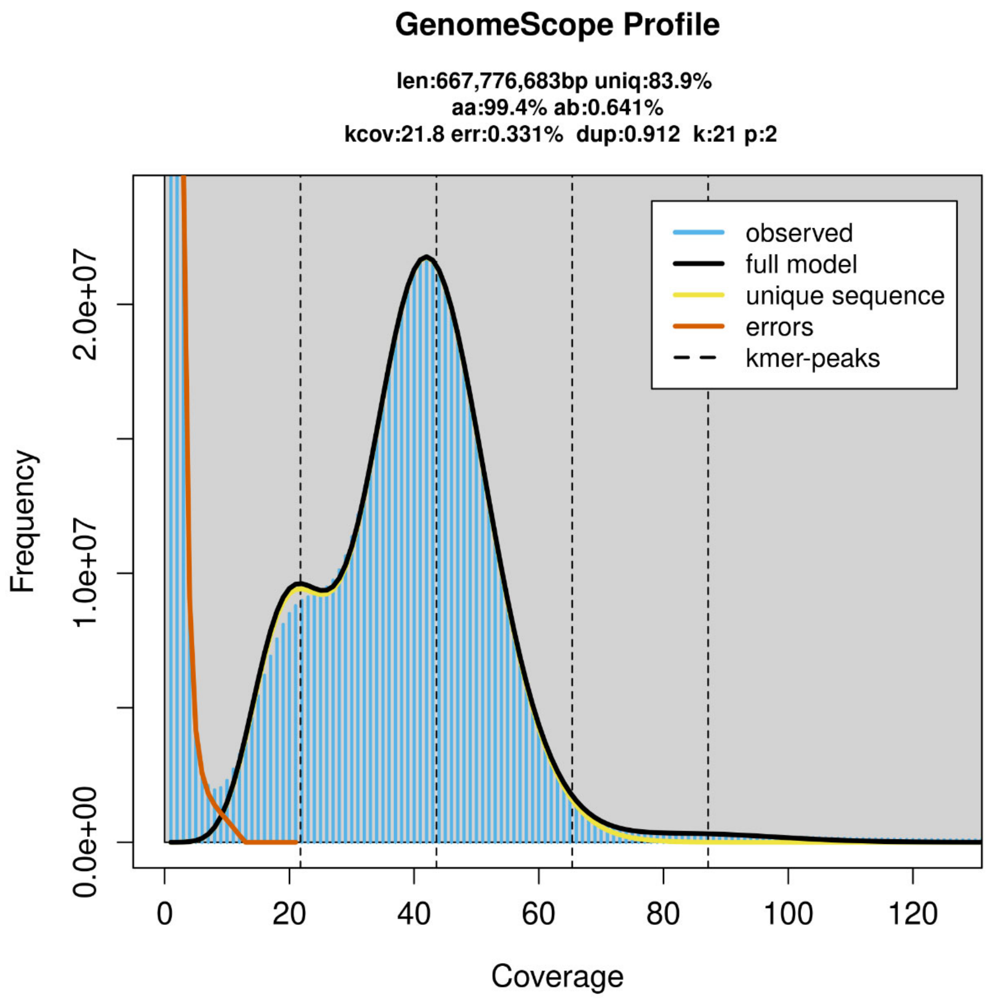

Supplementary Figure S1. The GenomeScope plot shows the distribution of 21-mer depth for NGS reads of *Tenualosa ilisha*. The x-axis displays the coverage depth, while the y-axis indicates the count of k-mers observed at each depth.

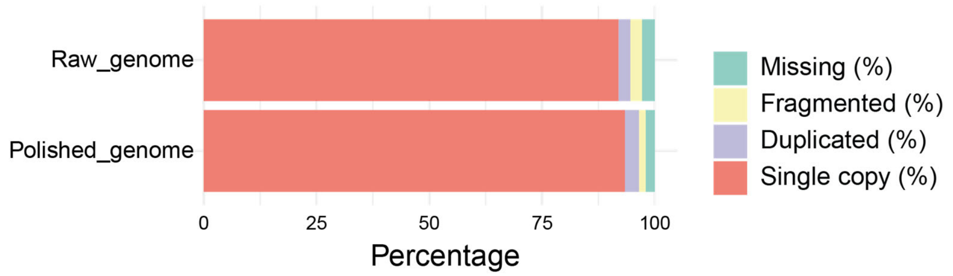

Supplementary Figure S2. The bar plot represents the BUSCO percentage of *Tenualosa ilisha* against the vertebrate databases

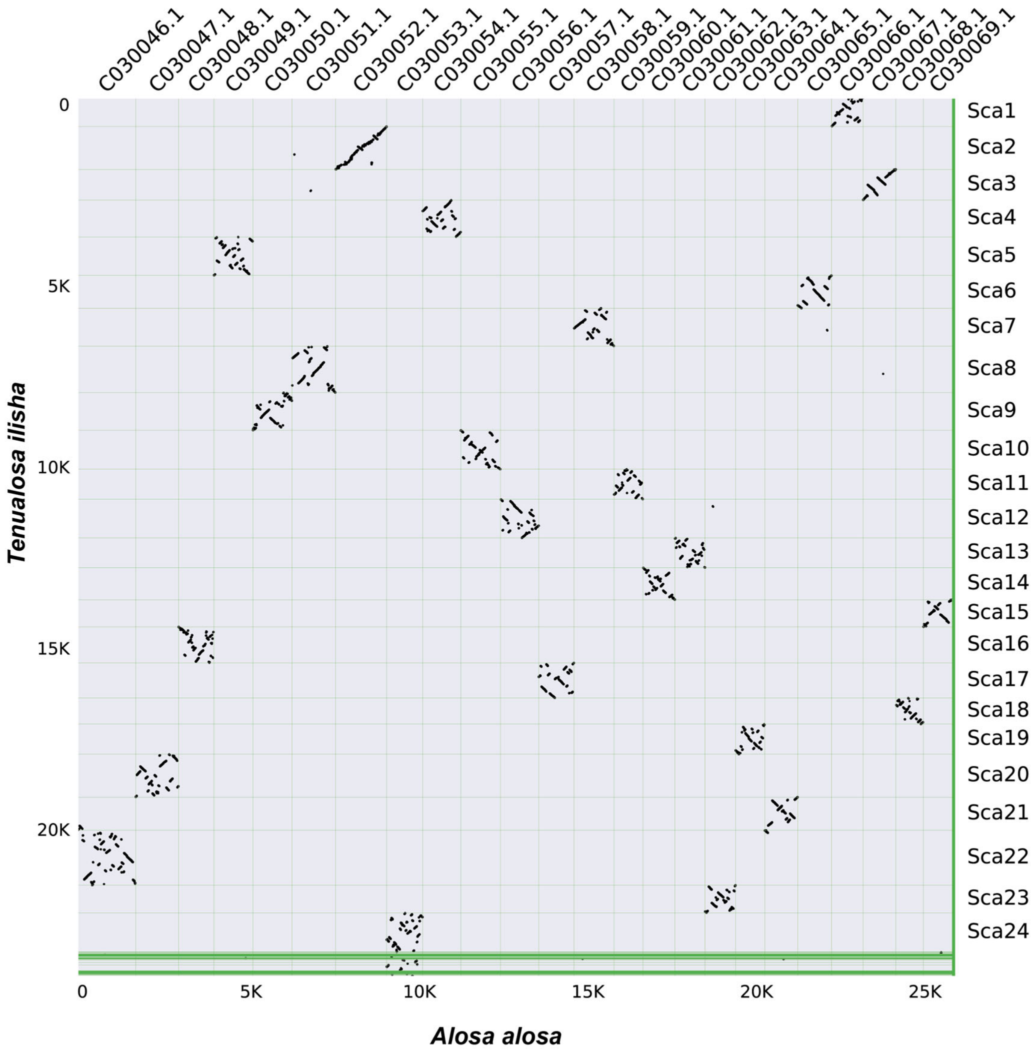

Supplementary Figure S3. The dot plot represents syntenic blocks shared between *Tenualosa ilisha* and *Alosa alosa*.

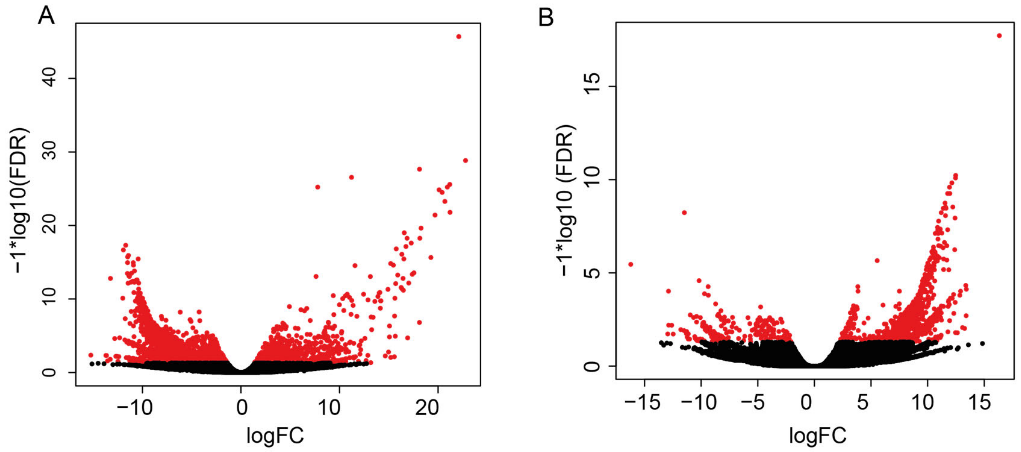

Supplementary Figure S4. Volcano plots representing transcripts with expression differences in the liver (A) and muscle (B) of *Tenualosa ilisha*. Red dots indicate transcripts with significant expression differences, while black dots represent non-significant transcripts.

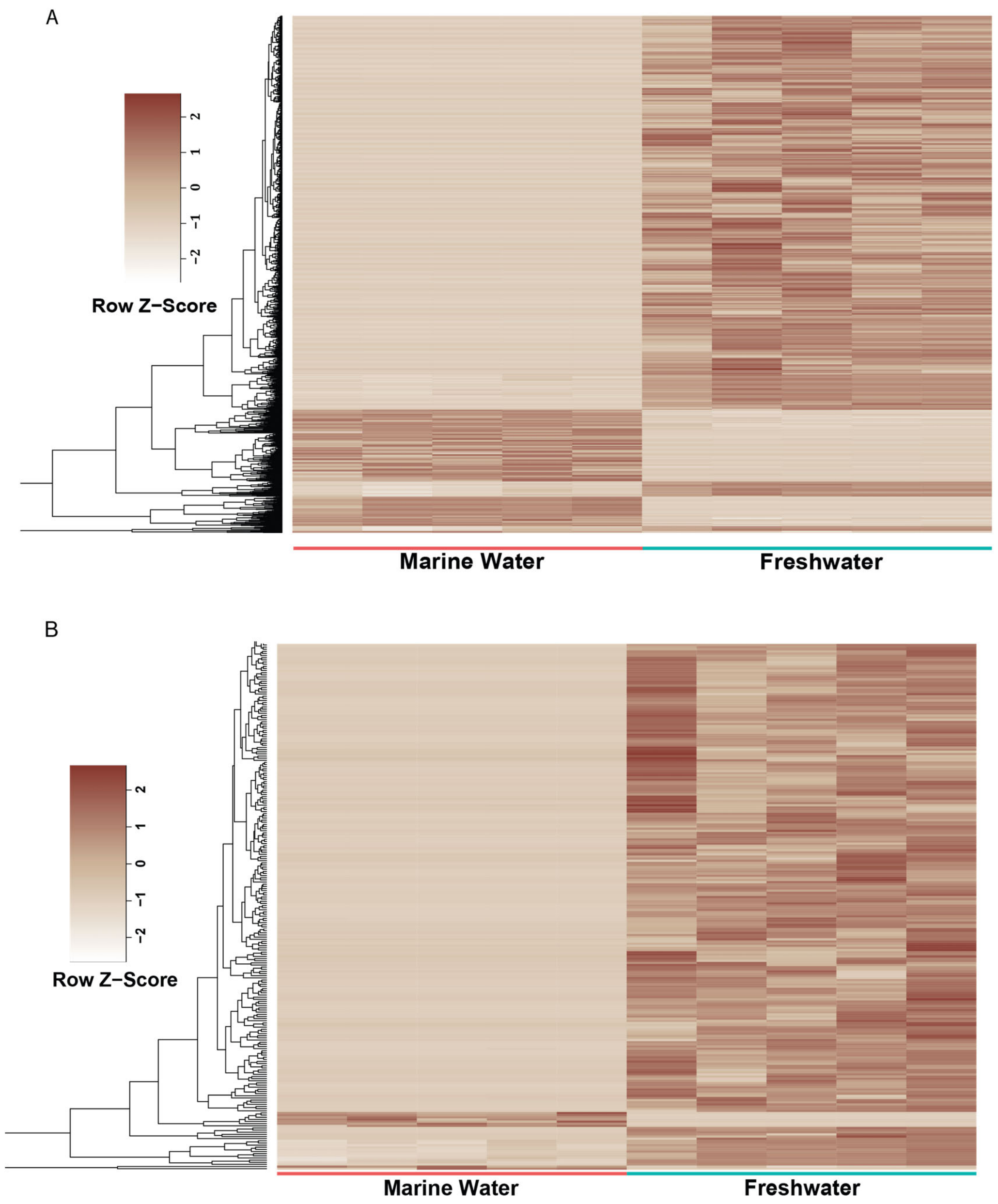

Supplementary Figure S5. Heatmap showing differentially expressed transcripts in the liver (A) and muscle (B) of *Tenualosa ilisha* with a log 4-fold change and a false discovery rate (FDR) of less than 0.001.

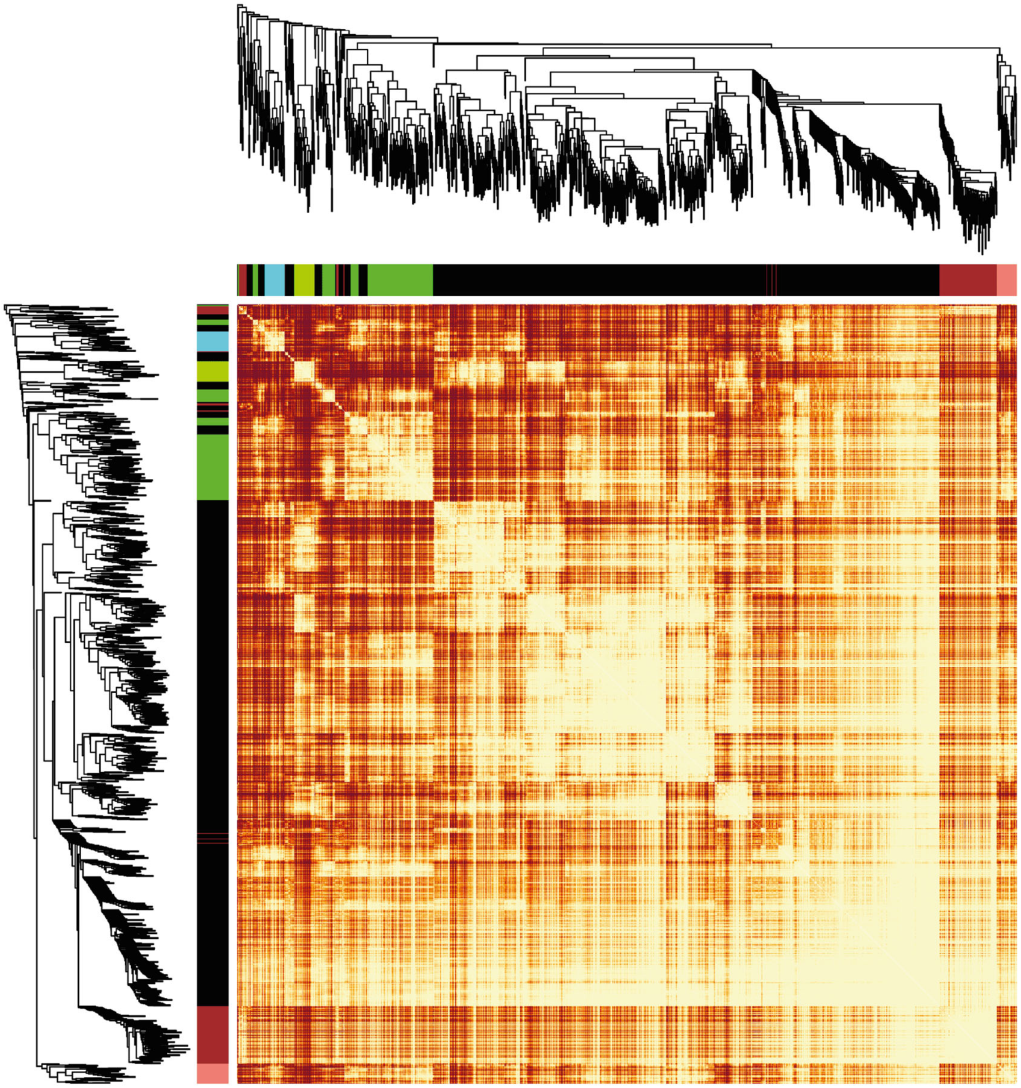

Supplementary Figure S6. Network heatmap for all DE transcripts from liver of *Tenualosa ilisha* taken for WCGNA analysis

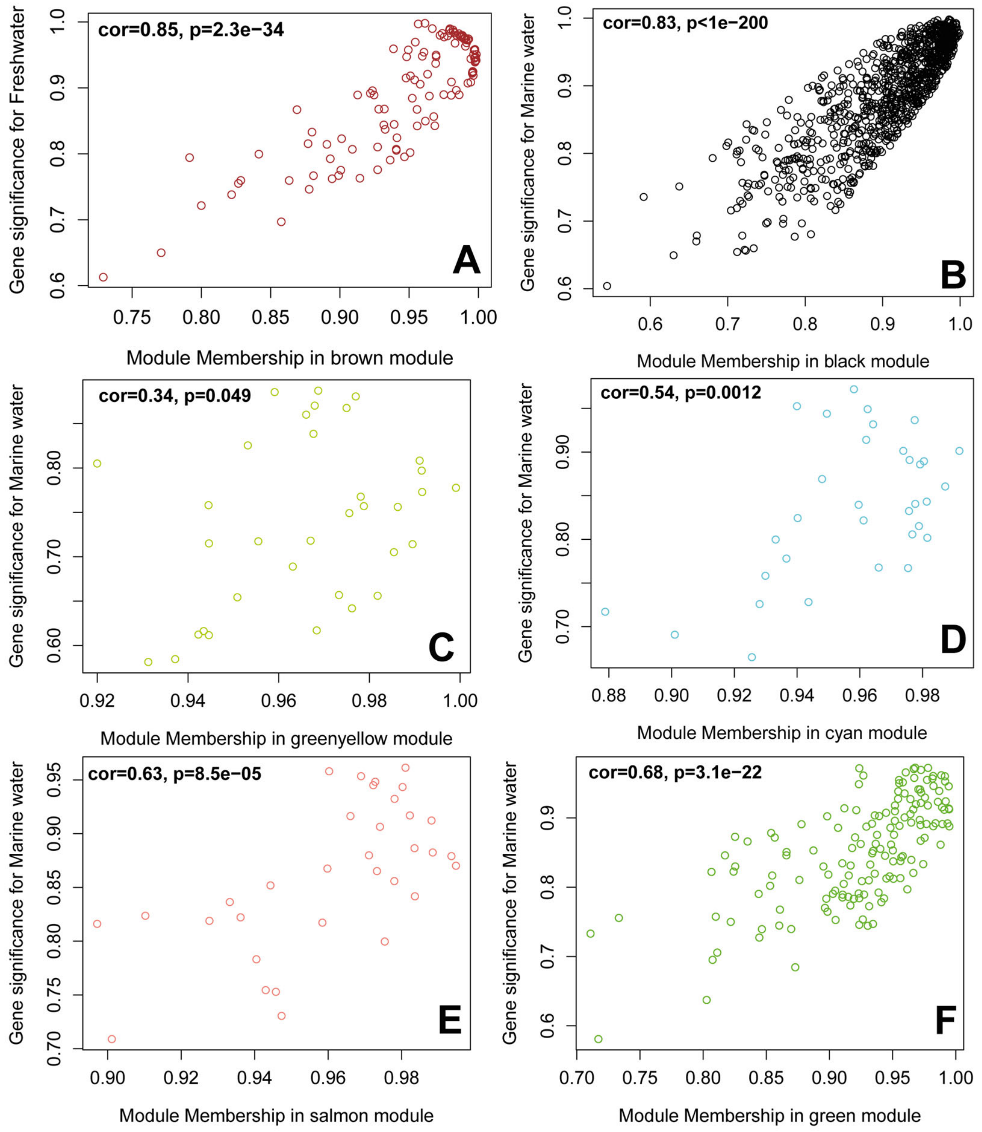

Supplementary Figure S7. Module membership vs. gene significance for liver transcripts of *Tenualosa ilisha*
A: Relationship between module membership and gene significance for the freshwater environment; B-E: Relationship between module membership and gene significance for marine water environment.

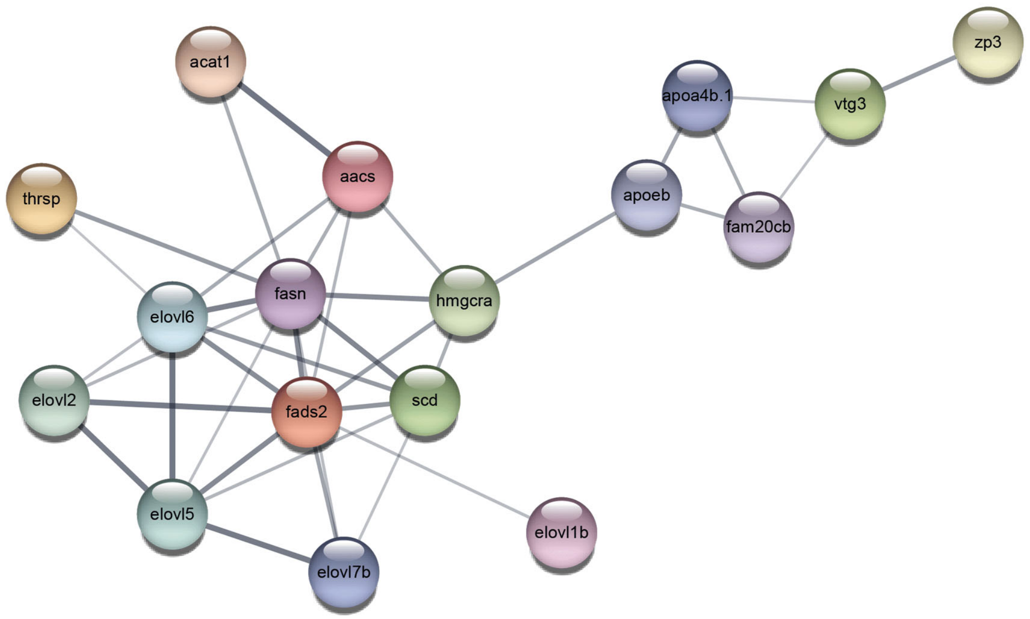

Supplementary Figure S8. String network connecting FA synthesis pathway with developmental associate genes in liver of *Tenualosa ilisha*

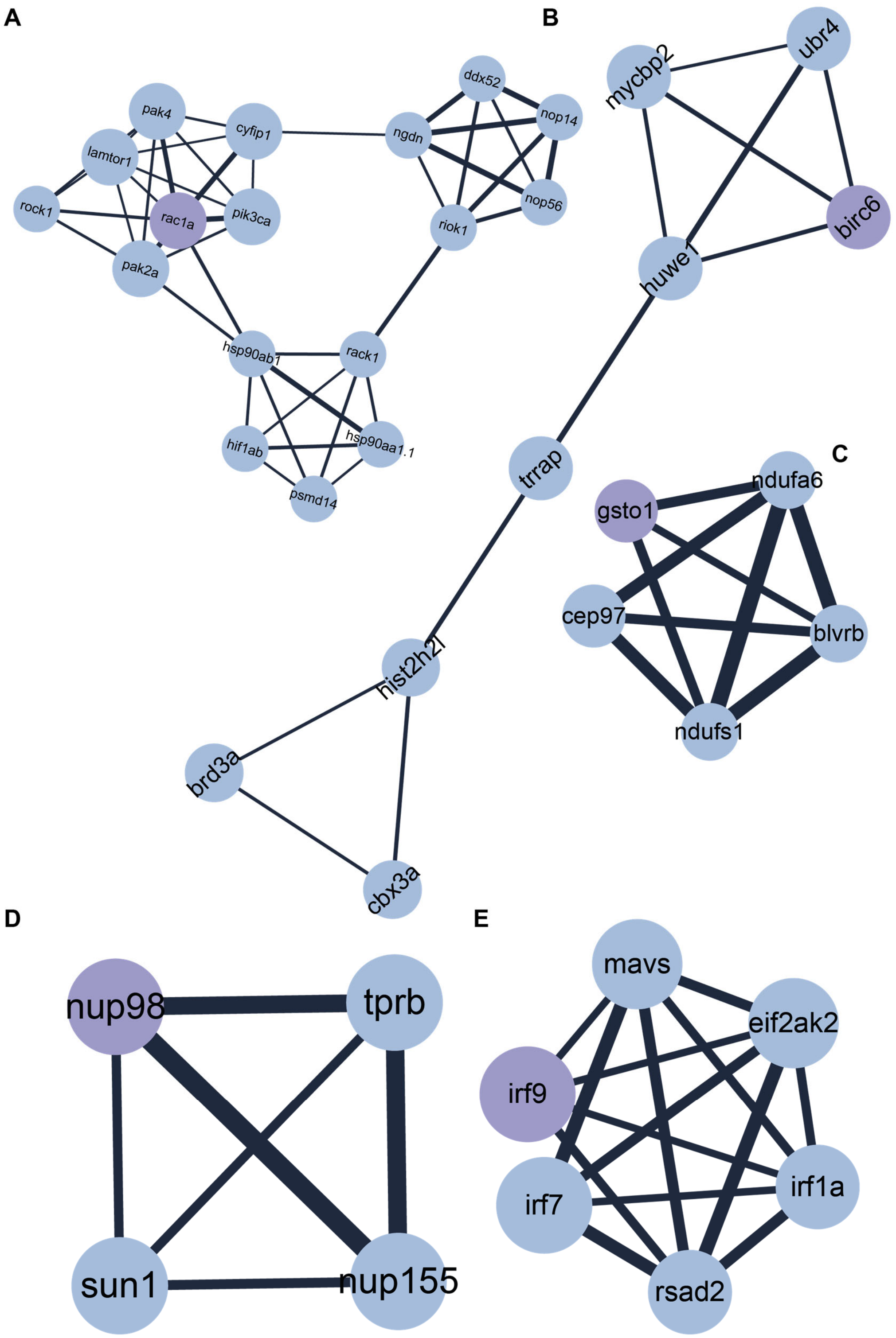

Supplementary Figure S9. (A-E) Protein-protein interactions of STRING database for marine water environments in liver of *Tenualosa ilisha*.

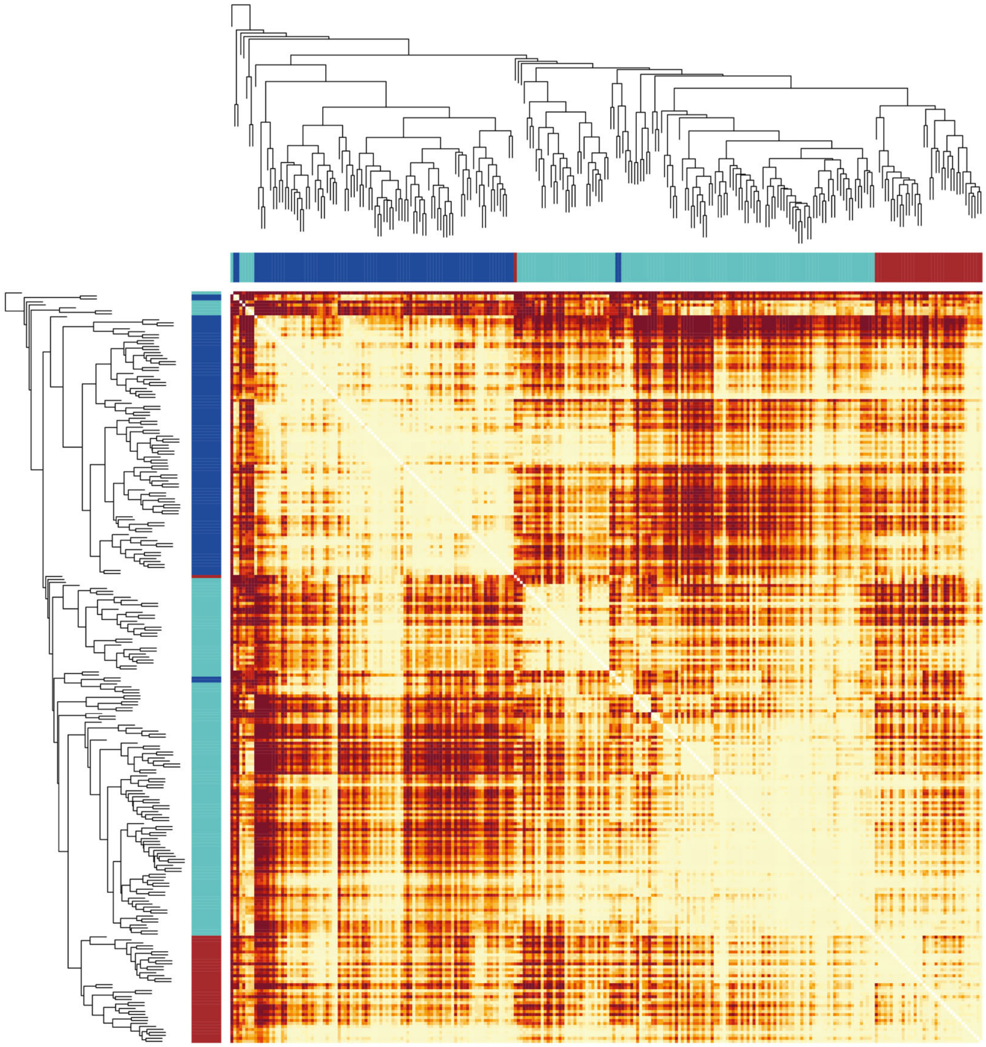

Supplementary Figure S10. Network heatmap for all DE transcripts from muscle of *Tenualosa ilisha* taken for WCGNA analysis

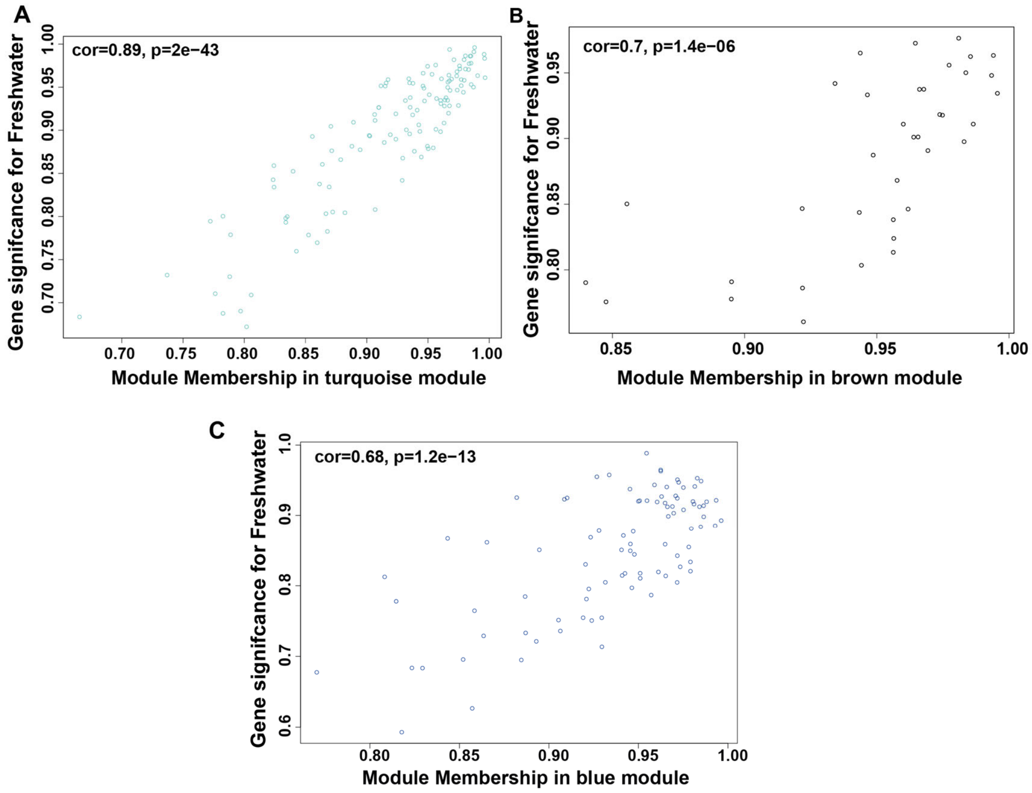

Supplementary Figure S11. Module membership vs. gene significance for muscle transcripts of *Tenualosa ilisha*. (A-C) Relationship between module membership and gene significance for the freshwater environment

Supplementary Table S1. Assembly statistics of *Tenualosa ilisha* genome

Chromosome Name	Size (MB)	Percentage (%)
Chr1	38.84	5.19
Chr2	32.85	4.39
Chr3	32.01	4.27
Chr4	31.55	4.21
Chr5	31.21	4.17
Chr6	31.04	4.14
Chr7	30.19	4.03
Chr8	30.12	4.02
Chr9	30.04	4.01
Chr10	29.8	3.98
Chr11	29.59	3.95
Chr12	29.36	3.92
Chr13	28.75	3.84
Chr14	28.72	3.83
Chr15	28.14	3.76
Chr16	27.91	3.73
Chr17	27.56	3.68
Chr18	27.11	3.62
Chr19	26.34	3.52
Chr20	25.97	3.47
Chr21	25.9	3.46
Chr22	25.49	3.40
Chr23	24.15	3.22
Chr24	24.13	3.22
Chromosome (%)	93.03%	
Genome Size	748993948	
Scaffold N50	29364864	
Contig N50	7136221	
N%	0.02%	
GapsPerGb	397	

Supplementary Table S2. Repeat statistics of the genome of *Tenualos ilisha*

Element Type	Number of Elements	Length Occupied (bp)	Percentage of Sequence (%)
Retroelements	205,383	62,506,091	8.35
SINEs:	60,330	20,504,944	2.74
Penelope	4,011	981,095	0.13
LINEs:	110,997	31,178,003	4.16
CRE/SLACS	0	0	0.00
L2/CR1/Rex	90,852	25,490,581	3.40
R1/LOA/Jockey	280	77,504	0.01
R2/R4/NeSL	5,342	797,872	0.11
RTE/Bov-B	4,964	670,517	0.09
L1/CIN4	3,841	849,391	0.11
LTR elements:	34,056	10,823,144	1.45
BEL/Pao	2,660	758,622	0.10
Ty1/Copia	589	361,568	0.05
Gypsy/DIRS1	17,614	6,199,574	0.83
Retroviral	11,879	3,314,235	0.44
DNA transposons	194,587	36,104,470	4.82
hobo-Activator	64,400	11,547,666	1.54
Tc1-IS630-Pogo	24,438	6,288,716	0.84
En-Spm	0	0	0.00
MuDR-IS905	0	0	0.00
PiggyBac	3,126	1,031,900	0.14
Tourist/Harbinger	13,354	3,186,146	0.43
Other (Mirage, P-element, Transib)	484	253,032	0.03
Rolling-circles	13,725	2,919,766	0.39
Unclassified	563,560	84,741,009	11.31
Total interspersed repeats	1,833,515,70	24.48%	
Small RNA	4,664	725,766	0.10
Satellites	636	488,885	0.07
Simple repeats	898,755	47,101,545	6.29
Low complexity	81,795	6,061,353	0.81

Supplementary Table S3. Top 10 enriched GO terms over the positively selected genes (Biological process) in *Tenualosa ilisha*

Term	No. of genes	Background Gene	P-value	FDR	Description
GO:0008152	312	9636	2.49e-28	2.58e-24	Metabolic process
GO:0006807	270	7706	5.32e-28	2.75e-24	Nitrogen compound metabolic process
GO:0044238	283	8363	2.04e-27	7.05e-24	Primary metabolic process
GO:0071704	294	8915	4.36e-27	1.13e-23	Organic substance metabolic process
GO:0043170	251	6981	6.81e-27	1.41e-23	Macromolecule metabolic process
GO:0044237	276	8460	8.90e-24	1.54e-20	Cellular metabolic process
GO:0009987	461	19185	2.61e-21	3.86e-18	Cellular process
GO:0034641	136	3180	1.26e-18	1.63e-15	Cellular nitrogen compound metabolic process
GO:0090304	100	1940	1.41e-18	1.63e-15	Nucleic acid metabolic process
GO:0044260	194	5554	8.28e-18	8.57e-15	Cellular macromolecule metabolic process

Supplementary Table S4. Top 10 enriched GO terms over the positively selected genes (Cellular component) in *Tenualosa ilisha*

Term	No. of genes	Background Gene	P-value	FDR	Description
GO:0005622	448	17775	4.80e-25	7.07e-22	Intracellular
GO:0043226	405	15396	1.48e-22	8.94e-20	Organelle
GO:0043227	371	13433	1.21e-22	8.94e-20	Membrane-bounded organelle
GO:0043229	386	14521	6.05e-21	2.23e-18	Intracellular organelle
GO:0043231	339	12116	8.13e-20	2.40e-17	Intracellular membrane-bounded organelle
GO:0070013	146	3543	1.04e-18	2.55e-16	Intracellular organelle lumen
GO:0110165	504	23494	3.10e-15	5.07e-13	Cellular anatomical entity
GO:0032991	185	5510	4.12e-15	6.07e-13	Protein-containing complex
GO:0031981	118	2976	9.20e-14	1.23e-11	Nuclear lumen
GO:0005737	335	13295	1.17e-11	1.43e-09	Cytoplasm

Supplementary Table S5. Top 10 enriched GO terms over the positively selected genes (Molecular function) in *Tenualosa ilisha*

Term	No. of genes	Background Gene	P-value	FDR	Description
GO:0003824	216	7610	1.69e-10	5.24e-07	Catalytic activity
GO:0140097	21	193	1.07e-09	1.65e-06	Catalytic activity, acting on DNA
GO:0003723	53	1273	3.45e-07	0.00035	RNA binding
GO:0034061	7	22	9.59e-07	0.00074	DNA polymerase activity
GO:0005488	387	17458	4.52e-06	0.0028	Binding
GO:0005515	203	7972	5.23e-06	0.0028	Protein binding
GO:0140101	12	122	1.05e-05	0.0046	Catalytic activity, acting on a tRNA
GO:0019899	71	2207	2.82e-05	0.0109	Enzyme binding
GO:0003887	5	20	9.61e-05	0.033	DNA-directed DNA polymerase activity
GO:0032451	6	36	0.00014	0.0424	Demethylase activity
